# 

**Published:** 1900-12-01

**Authors:** 


### NOTICE TO CORRESPONDENTS.

All MSS., Letters, Books for Review, and other matters intended for the Editor should be addressed The Editor, "The Hospital," The Hospital Building, 28 & 29 Southampton Street, Strand, W.C. The Editor cannot undertake to return rejected MS., even when accompanied by stamped directed envelope.

**Notice to Advertisers and Subscribers.**

All Advertisements, Orders for Copies of The Hospital, and Business Communications should be addressed to **THE MANAGER (Not to the Editor),** The Hospital, 28 & 29 Southampton Street, Strand, London, W.C.

The Cost of Subscription, post free, is as follows:—Per week, 2½d.; per quarter, 2s. 8d.; per half-year, 5s. 3d.; per annum, 10s. 6d. Foreign:—Per annum, 15s. 2d., payable, in all cases, in advance.

**NOTICE.—Vol. XXVIII. of "The Hospital" (April to September, 1900), in handsome cloth binding, is now on sale at the Office of this Journal, price, 7s. 6d. Cases for binding the Numbers contained in this Volume 1s. 6d. Index to Vol. XXVIII., 2½d., post free.**

**The Monthly Part for December is now ready. Price 6d., by post 8d.**

### BOOKS RECEIVED.

Wodderspoon and Co.
"The Combined Case Book, with Temperature Charts." By S. G.

Longmans, Green and Co.
"The Present Position of the Treatment of Simple Fractures of the Limbs." By William H. Bennett, F.R.C.S.

Thomas Bennett.
"Consumption." By Thomas Bennett.

John Wright and Co.
"Golden Rules of Skin Practice." By David Walsh, M.D.

J. B. Baillière et Fils.

"Catalogue General, 1901."

Ames, Heller and Co., Perth.
"Baby Mine." By E. Paget Thurstan, M.D., B.A. Cantab.

### Scraps and Gleanings.

During the year ended September 29th, 15,519 patients were treated at the Derbyshire Royal Infirmary. The cost of maintenance during the year was £9,501, being £1,270 in excess of the ordinary income.

The first systematic collection from the working men of Coventry and Warwickshire on behalf of the Coventry and Warwickshire Infirmary was made in 1874, when the sum collected was £132 18s. This year it was £1,250, and during the 26 years since the Working Men's Fund was founded it has contributed altogether to the Infirmary the sum of £20,402.

The Birmingham Hospital Saturday collection for the present year amounted to £18,204, against £18,151 the previous year. Of this sum the Birmingham General Hospital received £3,100, the Queen's Hospital £2,000, the General Dispensary £1,000, and seventeen other institutions amounts totalling £3,900, making £10,000 distributed out of the £18,204 collected.

The matter of the proposed isolation hospital for Newhaven has been referred by the Newhaven Urban Council to the General Purposes Committee, with a view to their recommending that the City Council should be petitioned to constitute the urban district of Newhaven a hospital district under the Isolation Hospital Act, and also to obtain a contribution towards the erection and establishment expenses of an isolation hospital.

During the last nine months the subscriptions to the Newcastle Infirmary amounted to £3,296, as against £2,462 received during the corresponding portion of last year. The donations were £1,386, as against £330. Church and chapel collections were £376, as against £222. The increases in the aggregate were £2,070. It is intended to establish an out-patient department for mental diseases at the Newcastle Infirmary. The class of case to be treated is that of patients who are mentally afflicted, but whose ailments are not sufficient to warrant them being moved to an asylum.

An exhaustive history of the Norfolk and Norwich Hospital has been compiled by Sir Peter Eade, who has been closely connected with this hospital for upwards of 50 years, having entered it as a pupil in 1843, being elected physician to the hospital in 1858, senior physician in 1885, and consulting physician in 1888. Sir Peter Eade's work is divided into two parts, the first apparently consisting of selected extracts from the minute books and dealing with the history of the hospital from the first year of its opening in 1772. Part II. deals with the modern hospital from 1883 to 1900. The whole work is one of great interest to all to whom the history of Norfolk and Norwich appeals.

### The Student of Medicine.

### APPOINTMENTS.

**T. Aubrey,** M.R.C.S., L.R.C.P.Lond., appointed District Medical Officer of the Newmarket Union. **Thos. Irvine Bonner,** M.A., M.B., C.M.Aberd., appointed Medical Officer of Health to the Towyn Urban District Council. **Miss Ada M. Browne,** L.S.A., appointed Assistant Anæsthetist to the Chelsea Hospital for Women. **Charles E. Fenn,** M.B.Durh., M.R.C.S., L.R.C.P., appointed House-Surgeon to the Worcester Infirmary. **A. Falconer Hayden,** M.R.C.S.Eng., L.R.C.P.Lond., appointed House-Surgeon to the County Hospital, Newport, Monmouthshire. **J. S. Hunt,** M.R.C.S., appointed Medical Superintendent of the Dunwich Benevolent Asylum, and Medical Officer for the Care, Supervision, and Inspection of Lepers at Stradbroke Island, Queensland. **Herbert Jones,** L.R.C.S.I., L.S.A., D.P.H.Camb., appointed Medical Officer of Health for the Hereford Rural District Council. **Channing Neill,** M.D., appointed Medical Officer for Fidsvold, Queensland. **J. F. O'Meara,** L.R.C.P., L.R.C.S.Edin., appointed District Medical Officer of the Walsall Union. **W. F. Oyston,** M.B., Ch.B.Vict., appointed Certifying Factory Surgeon for the Osmotherly District. **R. G. Pollock,** M.R.C.S., L.R.C.P.Lond., appointed Medical Officer for the Northern District of the Godstone Union. **John A. Shaw-Mackenzie,** M.D.Lond., appointed Physician to Out-patients at the Samaritan Free Hospital for Women and Children. **John Steed,** M.D.Edin., appointed Medical Officer to Jarvis's Charity, Staunton-on-Wye. **P. J. W. Ternau,** L.R.C.P., L.R.C.S.I., appointed District Medical Officer of the Smallburgh Union. **Edwin Thomas Walker,** L.R.C.P.&S.Edin., L.F.P.&S.Glasg., appointed Medical Officer of Health for the Midgley Urban District. **J. A. Ward,** M.R.C.S., L.R.C.P.Lond., appointed Medical Officer to the Training Ship *Shaftesbury*, Grays. **G. S. Wild,** M.D.Durh., M.R.C.S.Eng., appointed Honorary Physician to the Bootle Borough Hospital.

